# Supplementary figures and images for: The magnitude of neonatal near miss and associated factors among live births in public hospitals of Jimma Zone, Southwest Ethiopia, 2020: A facility-based cross-sectional study
Source: PLoS One. 2021 May 14;16(5):e0251609. doi: 10.1371/journal.pone.0251609 (PMC8121534; doi:10.1371/journal.pone.0251609)

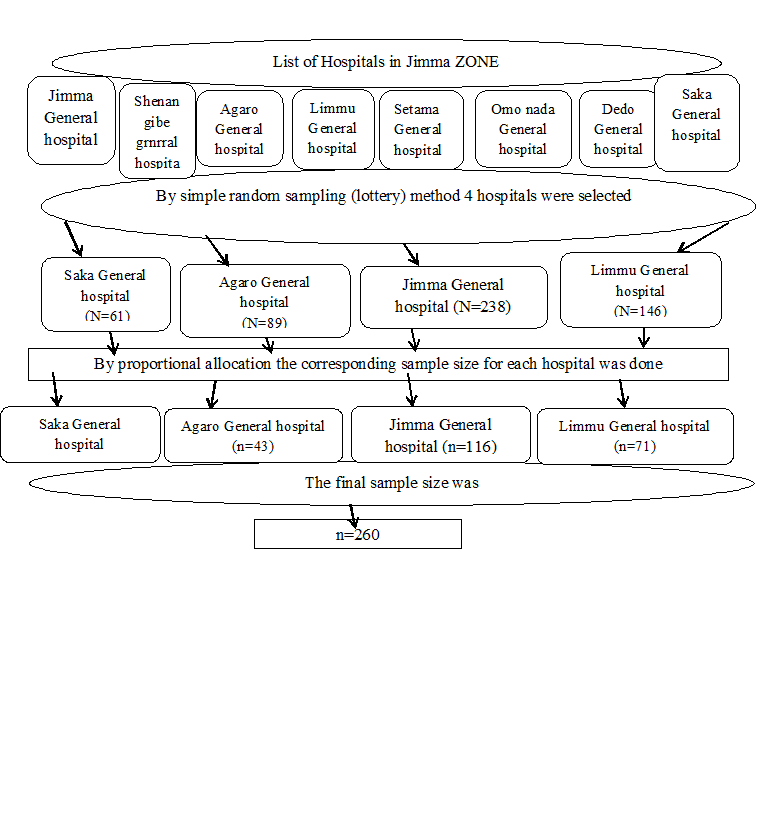

Supplement: S1 Fig — (TIF) [file pone.0251609.s001.tif]
