# Supplementary material for: The magnitude of neonatal near miss and associated factors among live births in public hospitals of Jimma Zone, Southwest Ethiopia, 2020: A facility-based cross-sectional study
Source: PLoS One. 2021 May 14;16(5):e0251609. doi: 10.1371/journal.pone.0251609 (PMC8121534; doi:10.1371/journal.pone.0251609)
Supplement: S1 Questionnaire — (DOCX) [file pone.0251609.s003.docx]

***Data collection tool for assessing the magnitude of neonatal near miss and associated factors among live births in selected public hospitals of Jimma zone, southwest Ethiopia,2020***

| **S No** | **Questions** | **Response** | **Code** |
| --- | --- | --- | --- |
| **Part I: Identifications** | | | |
| 101 | Hospital Name | 1. JMC 2. Agaro General Hospital 3. Saka district hospital 4. Limmu general hospital |  |
| 102 | Hospital code | **____/___/____** |  |
| 103 | Date form filled to start | ____/___/____ |  |
| 104 | Woreda /District | ________________________ |  |
| 105 | Kebele | ________________________ |  |
| 106 | Admission date | _____/____/____EC |  |
| 107 | Maternity record number/Medical | _________________ |  |
| **Part II Socio demographic and economic characteristics** | | | |
| 201 | How old are you? | _______ (in completed year) |  |
| 202 | What is your marital status? | 1. Married 2. Single 3. Widowed 4. Divorced |  |
| 203 | What is your ethnicity? | 1. Oromo 2. Dawuro 3. Amhara 4. Tigrie 5. Gurage 6. Others________________ |  |
| 204 | What is your educational status? | 1. No formal education 2. Primary (1-8) 3. Secondary (9-12) 4. College and above |  |
| 205 | What is paternal educational status? | 1. No formal education 2. Primary (1-8) 3. Secondary (9-12) 4. College and above |  |
| 206 | What is your religion? | 1. Orthodox 2. Catholic 3. Protestant 4. Muslim 5. Others |  |
| 207 | What is the occupation of the mother? | 1. Housewife 2. Merchant 3. Government employer 4. Daily laborer 5. E other |  |
| 208 | What is the occupation of the father? | 1. Farmer 2. Merchant 3. Government employer 4. private employer 5. Daily laborer |  |
| 209 | Where is the place of residence? | 1. Urban 2. Rural |  |
| **Part III: reproductive and obstetric related questions (Record review if it is available, if not interview the mother)** | | |  |
| 301 | Number of pregnancies (gravidity)? | __________ (in number) |  |
| 302 | Number of birth orders (parity)? | __________ (in number) |  |
| 303 | Do you have pervious history of still birth? | 1. Yes 2. No |  |
| 304 | Do you have pervious history of abortion? | 1. Yes 2. No |  |
| 305 | The duration between the current birth and the preceding birth in months? (birth interval) | __________ (in months) |  |
| 306 | Do you have previous history of neonatal death? | 1. Yes 2. No |  |
| 307 | Do you have previous history of preterm birth? |  |  |
| 401 | Did you received antenatal care in your last pregnancy? | 1. Yes 2. No |  |
| 402 | If yes, number of visits | _______________ |  |
| **Part V: Maternal Medical history during pregnancy and Obstetric related questions (Record review/interview)** | | |  |
| 501 | Date of delivery or end of pregnancy | ____/___/____ |  |
| 502 | Place of delivery |  |  |
| 503 | Mode of delivery | 1. Spontaneous vaginal delivery 2. Instrumental delivery 3. Caesarean section |  |
| 504 | Ante partum Hemorrhage | 1. Yes 2. No |  |
| 505 | Premature rupture of member | 1. Yes 2. No |  |
| 506 | Hypertension | 1. Yes 2. No |  |
| 507 | If, Yes | 1. Pre-eclampsia 2. Eclampsia 3. HELLP 4. Chronic hypertension 5. Gestational hypertension |  |
| 508 | Diabetes mellitus | 1. Yes 2. No |  |
| 509 | Infection | 1. Yes 2. No |  |
| 510 | If, Yes | 1. Unspecified infection 2. Puerperal endometritis 3. Pyelonephritis 4. Septicemia 5. Peritonitis 6. Parietal suppuration 7. Malaria 8. Syphilis 9. Other systemic infection |  |
| 511 | Dystocia | 1. Yes 2. No |  |
| 513 | If, Yes | 1. Uterine pre-rupture 2. Prolonged labor 3. Feto-pelvic disproportion |  |
| 514 | Other pathologies | 1. Yes 2. No |  |
| 515 | If, Yes | 1. HIV/AIDS 2. Embolic diseases  (thrombosis/amniotic fluid or gaseous embolism 3. Heart disease 4. Sickle-cell disease 5. Other (specify: __________) |  |
|  | **Newborn related characteristics** | |  |
| **516** | Presentation | 1. Cephalic 2. Breech 3. Transverse/face/brow 4. Other, specify__________ |  |
| **517** | Sex | 1. Male 2. Female |  |
| 518 | Admitted to special care or intensive care unit | 1. Yes 2. No |  |

**Identification criteria for neonatal near miss conditions**

| **Part VI: Neonatal Near-Miss Criteria’s** | | |
| --- | --- | --- |
| **Pragmatic Markers** | | |
| 601 | Gestational age | ______(in week) |
| 602 | Birth weight | ______(g) |
| 603 | Apgar score at 5th min | _______ |
| **Management Severity Criteria’s** | | |
| 604 | Use of intravenous antibiotics up to 7 days and before 28 days | 1. Yes 2. No |
| 605 | Nasal CPAP | 1. Yes 2. No |
| 606 | Any intubation | 1. Yes 2. No |
| 607 | Use of phototherapy in the first 24 hour | 1. Yes 2. No |
| 608 | Cardio pulmonary resuscitation | 1. Yes 2. No |
| 609 | Use of any vasoactive drug | 1. Yes 2. No |
| 610 | Use of anticonvulsants | 1. Yes 2. No |
| 611 | Use of surfactant | 1. Yes 2. No |
| 612 | Transfusion of blood derivatives | 1. Yes 2. No |
| 613 | Use of corticosteroid for treatment of refractory hypoglycemia | 1. Yes 2. No |
| 614 | Any surgical procedure | 1. Yes 2. No |
| 615 | Use of antenatal steroid | 1. Yes 2. No |
| 616 | Parenteral nutrition | 1. Yes 2. No |
| 617 | Congenital malformation – ICD-10 | 1. Yes 2. No |
| 618 | Admission to NICU | 1. Yes 2. No |
| **Is the newborn considered near-miss?** | | **a. Yes b. No** |
