# Supplementary material for: The magnitude of neonatal near miss and associated factors among live births in public hospitals of Jimma Zone, Southwest Ethiopia, 2020: A facility-based cross-sectional study
Source: PLoS One. 2021 May 14;16(5):e0251609. doi: 10.1371/journal.pone.0251609 (PMC8121534; doi:10.1371/journal.pone.0251609)
Supplement: Attachment — Submitted filename: Response to Reviewers.docx [file pone.0251609.s004.docx]

**AUTHORS’ RESPONSE TO ACADEMIC EDITOR AND REVIEWERS**

1. **AUTHOR’S RESPONSE TO EDITOR**

**Dear: Kazumichi Fujioka (Academic Editor)**

We thank you for a thorough reading and constructive comments and suggestions of our manuscript and for the opportunity to revise and resubmit. We are pleased to submit the revised research article “**The Magnitude of Neonatal Near Miss and Associated Factors among Live Births in Public Hospitals of Jimma Zone, Southwest Ethiopia, 2020: A Facility-based cross-sectional study**” for your consideration in the special collection of Plos One. On the following pages, you will find our response to the editor’s comments. On behalf of my co-authors, I thank you for your consideration of this resubmission. We appreciate your time and look forward to your response.

Sincerely,

Aklilu Habte(MPH)(corresponding author)

[**aklilihabte57@gmail.com**](mailto:aklilihabte57@gmail.com)

**AUTHORS’ RESPONSE TO ACADEMIC EDITOR**

PONE-D-21-03070

The Magnitude of Neonatal Near Miss and Associated Factors among Live Births in Public Hospitals of Jimma Zone, Southwest Ethiopia, 2020: A Facility-based cross-sectional study

PLOS ONE

Dear Dr. Hailegebireal,

Thank you for submitting your manuscript to PLOS ONE. After careful consideration, we feel that it has merit but does not fully meet PLOS ONE’s publication criteria as it currently stands. Therefore, we invite you to submit a revised version of the manuscript that addresses the points raised during the review process.

**Comment1:** The statistical concern arisen from our statistical editor. Please clarify these concerns pointed out.

**Response:** We appreciate and share the statistical editor's comment and suggestion, especially about sample size determination, and have now attempted to make it more clear in the "sample size determination" section of the revised manuscript with track changes," Page 4, Lines 110-119.

The contents and purpose is well understood.

Please submit your revised manuscript by Jun 04 2021 11:59PM. If you will need more time than this to complete your revisions, please reply to this message or contact the journal office at plosone@plos.org. When you're ready to submit your revision, log on to https://www.editorialmanager.com/pone/ and select the 'Submissions Needing Revision' folder to locate your manuscript file.

Please include the following items when submitting your revised manuscript:

•	A rebuttal letter that responds to each point raised by the academic editor and reviewer(s). You should upload this letter as a separate file labeled 'Response to Reviewers'.

•	A marked-up copy of your manuscript that highlights changes made to the original version. You should upload this as a separate file labeled 'Revised Manuscript with Track Changes'.

•	An unmarked version of your revised paper without tracked changes. You should upload this as a separate file labeled 'Manuscript'.

If you would like to make changes to your financial disclosure, please include your updated statement in your cover letter. Guidelines for resubmitting your figure files are available below the reviewer comments at the end of this letter.

PLOS ONE

**Response:** We appreciate your detailed reading of our manuscript and insightful comments and suggestions, as well as the opportunity to review and resubmit it. In the following sections, we have attempted to respond to all of the comments made by the editors and reviewers.

**Journal Requirements:**

**Comment1:** Please ensure that your manuscript meets PLOS ONE's style requirements, including those for file naming. The PLOS ONE style templates can be found at https://journals.plos.org/plosone/s/file?id=wjVg/PLOSOne_formatting_sample_main_body.pdf and <https://journals.plos.org/plosone/s/> file? Id=ba62/PLOS One_ formatting_ sample_ title_authors_affiliations.pdf

**Response:** Thank you for your thoughtful suggestion, and we do so according to the requirements of the journal.

**Comment2:** We suggest you thoroughly copyedit your manuscript for language usage, spelling, and grammar. If you do not know anyone who can help you do this, you may wish to consider employing a professional scientific editing service.

Whilst you may use any professional scientific editing service of your choice, PLOS has partnered with both American Journal Experts (AJE) and Editage to provide discounted services to PLOS authors. Both organizations have experience helping authors meet PLOS guidelines and can provide language editing, translation, manuscript formatting, and figure formatting to ensure your manuscript meets our submission guidelines. To take advantage of our partnership with AJE, visit the AJE website (http://learn.aje.com/plos/) for a 15% discount off AJE services. To take advantage of our partnership with Editage, visit the Editage website (www.editage.com) and enter referral code PLOSEDIT for a 15% discount off Editage services. If the PLOS editorial team finds any language issues in text that either AJE or Editage has edited, the service provider will re-edit the text for free.

**Response:** Thank you for your insightful comments and suggestions, which we found to be extremely helpful in enhancing the readability of our work. We consulted instructors from our university's language and literature departments to give due weight to grammar and typological issues, as per your suggestion. We also attempted to resolve the concerns posed in the manuscript's language, spelling, and grammar usage by doing so. Throughout the "revised manuscript with track changes," all of the amendments were highlighted.

Upon resubmission, please provide the following:

•The name of the colleague or the details of the professional service that edited your manuscript

•A copy of your manuscript showing your changes by either highlighting them or using track changes (uploaded as a *supporting information* file)

•A clean copy of the edited manuscript (uploaded as the new *manuscript* file)

**Comment3:** Please include in your Methods section (or in Supplementary Information files) the participating hospitals/ institutions. Furthermore, please provide additional details on the participant recruitment, proceeds including the inclusion and exclusion criteria used.

**Response:** Thank you so much for your helpful comment, and as per your suggestion, we have attempted to integrate all of the issues in the supporting information section of the submission process as S1-Fig1. Besides, we tried to make the Inclusion and exclusion criteria section more clear and highlighted it in the “population of the study” section of the “revised manuscript with track changes,” Page 4, Line 104-106.

**Comment4:** Finally, please clarify how informed ascent was documented for participants under the age of 18 years (ie written or verbal, if verbal please stated how verbal consent was recorded).

**Response:** Thank you for suggesting that the process of obtaining assent be made more clear. We kindly assure you that we took the written informed ascent, which we have now corrected and highlighted in the “Ethical approval and consent to participate” section of the “revised manuscript with track changes”, Page 7, Line 193.

__________________________________________________________________________________________________________END________________________________________

THANK YOU!!!

**AUTHORS’ RESPONSE TO REVIEWERS**

**DEAR: REVIEWERS**

We thank you for a thorough reading and constructive comments and suggestions of our manuscript and for the opportunity to revise and resubmit. We are pleased to submit the improved research article **“The Magnitude of Neonatal Near Miss and Associated Factors among Live Births in Public Hospitals of Jimma Zone, Southwest Ethiopia, 2020: A Facility-based cross-sectional study”** for your consideration in the special collection of Plos One. On the following pages, you will find our response to the reviewers’ comments. On behalf of my co-authors, I thank you for your consideration of this resubmission. We appreciate your time and look forward to your response independently as for reviewers 1, 2, and 3 respectively.

Sincerely,

Aklilu H. (MPH) (corresponding author)

[**aklilihabte57@gmail.com**](mailto:aklilihabte57@gmail.com)

**AUTHORS’ RESPONSE TO REVIEWER**S

1. Is the manuscript technically sound, and do the data support the conclusions?

The manuscript must describe a technically sound piece of scientific research with data that supports the conclusions. Experiments must have been conducted rigorously, with appropriate controls, replication, and sample sizes. The conclusions must be drawn appropriately based on the data presented.

Reviewer #1: Yes

Reviewer #2: Yes

Reviewer #3: Partly

**Response:** We appreciate the reviewers' positive feedback about the credibility of our work, and we have attempted to improve the revised version of the manuscript by responding to your comments and suggestions.

2. Has the statistical analysis been performed appropriately and rigorously?

Reviewer #1: Yes

Reviewer #2: I Don't Know

Reviewer #3: No

**Response:** We appreciate the reviewer's feedback on the analysis's appropriateness and rigor. All the details regarding the statistical analysis were described and highlighted in the “data analysis” section of the revised manuscript with track changes”, Page6, Line152-164.

3. Have the authors made all data underlying the findings in their manuscript fully available?

The PLOS Data policy requires authors to make all data underlying the findings described in their manuscript fully available without restriction, with rare exceptions (please refer to the Data Availability Statement in the manuscript PDF file). The data should be provided as part of the manuscript or its supporting information, or deposited to a public repository. For example, in addition to summary statistics, the data points behind means, medians, and variance measures should be available. If there are restrictions on publicly sharing data—e.g. participant privacy or use of data from a third party—those must be specified.

Reviewer #1: Yes

Reviewer #2: Yes

Reviewer #3: No

**Response:** Thank you for your suggestion, and we have included all of the data collection tools and data sets that support the current study's findings in the supporting information section as **“S1-data collection tool for MNM”**, **“S1-Data set for MNM”** and **“S1-Fig1”**.

4. Is the manuscript presented in an intelligible fashion and written in Standard English?

PLOS ONE does not copyedit accepted manuscripts, so the language in submitted articles must be clear, correct, and unambiguous. Any typographical or grammatical errors should be corrected at revision, so please note any specific errors here.

Reviewer #1: Yes

Reviewer #2: Yes

Reviewer #3: Yes

__________________________________________________________________________

**Response:** Thank you for your kind words about the way the information in the manuscript was presented.

**AUTHORS’ RESPONSE TO REVIEWERs**#**1**

**Comment1:** Good start for the unusual topic, I would suggest to have a wider study covering bigger population with different ethnic groups and socioeconomic status, Antenatal care and follow up is crucial in such study and has to be focused on.

**Response:** We appreciate your feedback and suggestions, and we will work to implement them in the future by conducting a large-scale study. We'd like to let you know that we've attempted to assess the level of ANC uptake in terms of frequency, and we've attempted to report the results on Page 9, Table 2 of the "Manuscript".

**AUTHORS’ RESPONSE TO REVIEWERs**#**2**

**Comment:** this article is very valuable because Neonatal Near Miss (NNM) data has not been collected in developing countries. Sample size is very large (260 cases) and very precisely analysed. Even though NNM is due to only the maternal pregnancy conditions (social factors are very important), the value of this study won't fade.

**Response:** thank you very much for your kind word regarding the value of our work to the scientific community.

**AUTHORS’ RESPONSE TO REVIEWERs#3**

Reviewer #3: This manuscript reports the percentage of neonatal near miss and investigates the associated risk factors in public hospitals of Jimma Zone, Southwest Ethiopia with a facility-based cross-sectional study in 2020. I have below comments.

**Comment1:** For the sample size, with the given assumptions (the prevalence of NNM of 50%, 5% margins of error), the sample size should have been 264 to guarantee the width of 95% confidence interval within +/-5% (corresponding to the planed 5% margins of error). With 10% non-response rate, the final sample size of 293 should have been planned. Please explain the discrepancy sample size calculation. N=255 to 260 would be good for a prevalence of NNM of ~20%. From the results on page 10, the NNM% in this study area was 26.7%. Is it a post-study calculation for the 5% margins of error?

**Response:** First, we'd like to thank you for your thorough review of our work, and we apologise for any errors in our reporting of what we were doing during sample size determination. To make things clearer, we attempted to depict the sample size estimation process as follows. Initially, a sample size of 423 was calculated using the following parameters: a 50% prevalence of NNM (because no similar research had been conducted in Ethiopia), a 95% confidence level, 5% margins of error, and a 10% non-response rate. Since the total population is less than 10,000 (N=534), we used the finite population correction formula. The total population of N=534 was obtained by averaging the trends of the previous three years' client flow for each of the selected hospitals in April and May. The more clarified statement regarding the sample size calculation was mentioned and highlighted in the "sample size determination" section of the “revised manuscript with track changes” Page 4, Lines 110-119.

**Comment2:** From the results on page 10, the NNM% in the study area was 26.7% and (95%CI: 21.6%-32.5). Please add % after 32.5.

**Response:** Thank you for the comment and we have corrected it accordingly and highlighted it on Page11, Line224 of the “revised manuscript with track changes”

**Comment3:** Are the selected four hospitals comparable on clinical function and the level of health care? Is there any potential cluster effect by hospital? If yes, the cluster effect needs to be considered in the logistic regression.

**Response:** We appreciate your thoughtful comment and concern. That was our initial concern as well, but we'd like to inform you that there were no differences in clinical function or health-care quality between hospitals. All of the hospitals that were chosen provided maternal and child health services based on national standards. As a result, the cluster effect was not taken into account during the logistic regression analysis.

**6**. PLOS authors have the option to publish the peer review history of their article (what does this mean?). If published, this will include your full peer review and any attached files.

If you choose “no”, your identity will remain anonymous but your review may still be made public.

**Response:** No

__________________________________________________________________________________________________________END________________________________________

THANK YOU!!!
